# Dataset on the effect of hardwood biochar on soil gravimetric moisture content and nitrate dynamics at different soil depths with FTIR analysis of fresh and aged biochar

**DOI:** 10.1016/j.dib.2019.104073

**Published:** 2019-05-28

**Authors:** Emily J. Cole, Omid R. Zandvakili, Baoshan Xing, Masoud Hashemi, Stephen Herbert, Hamid H. Mashayekhi

**Affiliations:** a Department of Environmental Science, Westfield State University, Westfield, MA 01086, USA; b Stockbridge School of Agriculture, Univ. of Massachusetts Amherst, Bowditch Hall, Amherst, MA 01003, USA; c Stockbridge School of Agriculture, Univ. of Massachusetts Amherst, Paige Lab, Amherst, MA 01003, USA

## Abstract

The goal of this research work was to determine widespread impact kiln-produced hardwood biochar has upon temperate agricultural soil characteristics in a long-term field experiment. This dataset is supplementary to the submitted research by [1] and presents select physical and chemical characteristics of the biochar and field plots amended with hardwood biochar. Data on soil gravimetric moisture content (GMC), soil acidity and soil nitrate-N concentration at lower depth of soil under different biochar application rates is presented. Fourier Transform Infrared (FTIR) spectroscopy is provided to demonstrate the difference between fresh and aged biochar in terms of surface functional group content.

**Table:** Specifications Table

Subject area	Soil-Agriculture
More specific subject area	Biochar, soil moisture content, nitrate-N leaching, FTIR
Type of data	Table, Graph
How data was acquired	Nitrate: QuickChem 8000, LaChat Instruments Al: MP-AES Agilent 4100 FTIR: PerkinElmer Spectrum One with ATR attachment, 2 cm^−1^ resolution
Data format	Raw, analyzed
Experimental factors	biochar sourced from sugar maple charcoal “fines” produced via cement kiln, slow pyrolysis, operating temperature of approximately 450C biochar and soil samples extracted using modified morgans solution
Experimental features	Gravimetric moisture content of control and biochar amended soils provided. Nitrate and Al content data of biochar and field soil samples as amended with biochar at rates of 0, 2, 4, 6, and 8% (w/w) provided along with characterized FTIR of fresh and aged biochar sampled from field.
Data source location	University of Massachusetts Amherst Crops and Animal Research and Education Farm in South Deerfield, MA.
Data accessibility	Data are accessible with the article
Related research article	The associated research article to this data set is [1].

**Table:** 

**Value of the data** • This data provides gravimetric moisture content of control (0% biochar) and biochar amended soils (2, 4, 6, and 8% w/w) revealing significant differences. • The data also provides information on nitrate retention at multiple depths of soil, 20–40, and total 0–40 cm depth. • This data presents biochar-amended soil content of Al and the corresponding soil pH over a three year-longitudinal field experiment. • FTIR spectra of both fresh and aged biochar presented showing changes to biochar functional groups through aging in the field.

## 1 Data

**Fig. 1:**
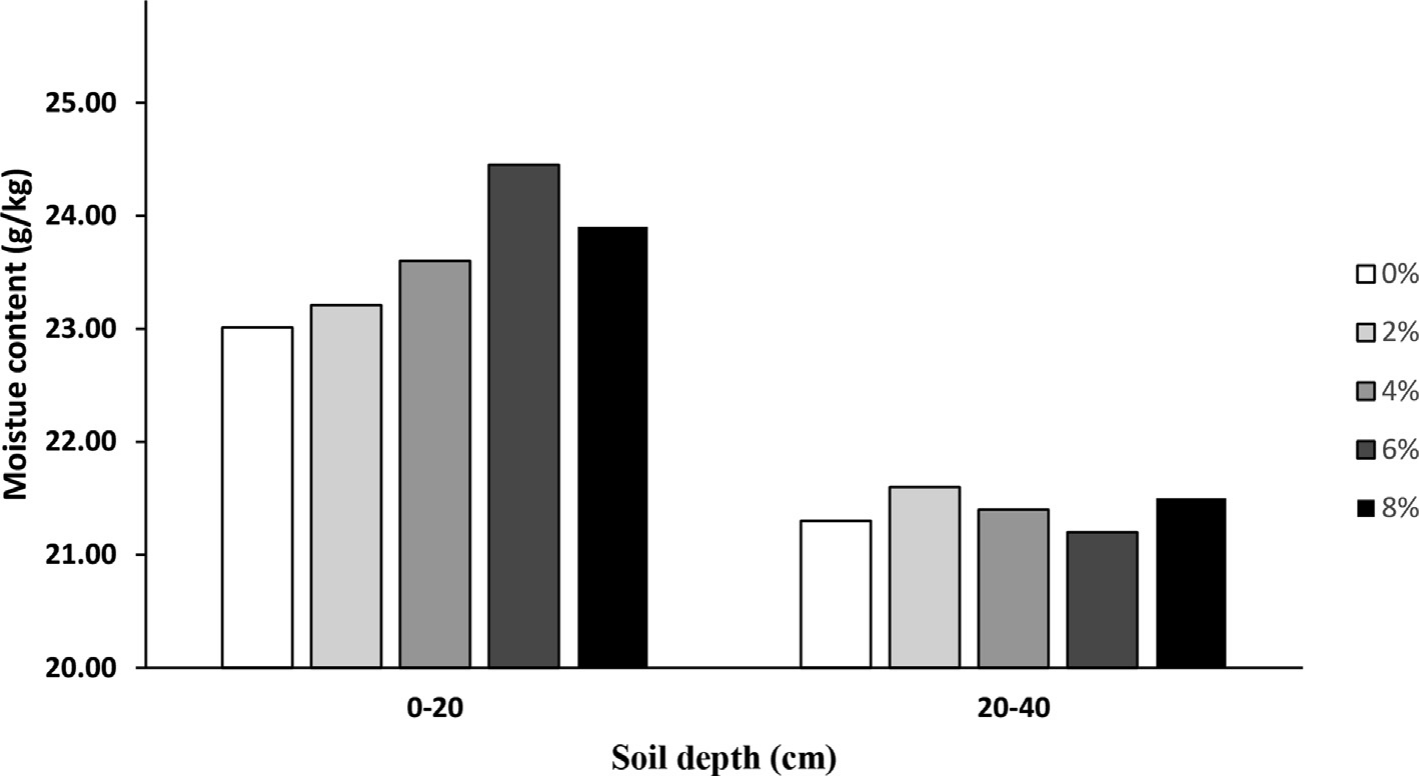
Gravimetric moisture content of biochar amended field plots.

The biochar used was investigated for changes in the gravimetric moisture content 48 hours after a rain event (Fig. 1). Differences were not significant for biochar treatment levels at the 20–40cm depth, however gravimetric moisture content increased significantly at the 0–20cm soil depth (p < 0.05).

**Fig. 2:**
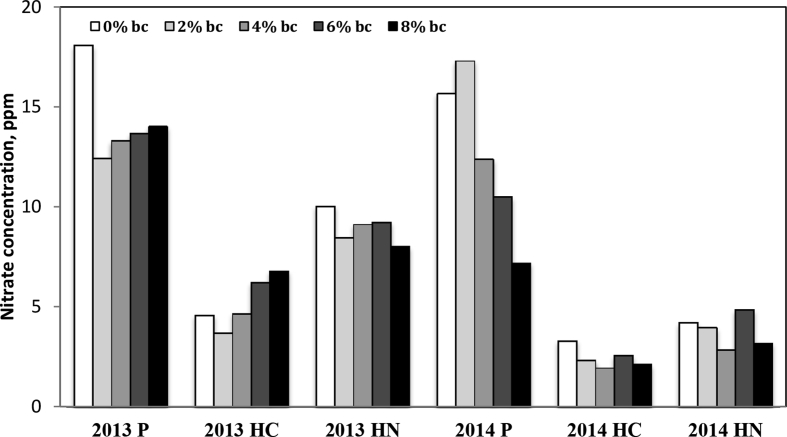
Soil nitrate concentration at PSNT and harvest, 20–40 cm depth. P is PSNT timing, HC is at time of harvest control (no fertilizer), HN is at time of harvest with nitrogen fertilizer treatment (56kg ha^−1^).

**Fig. 3:**
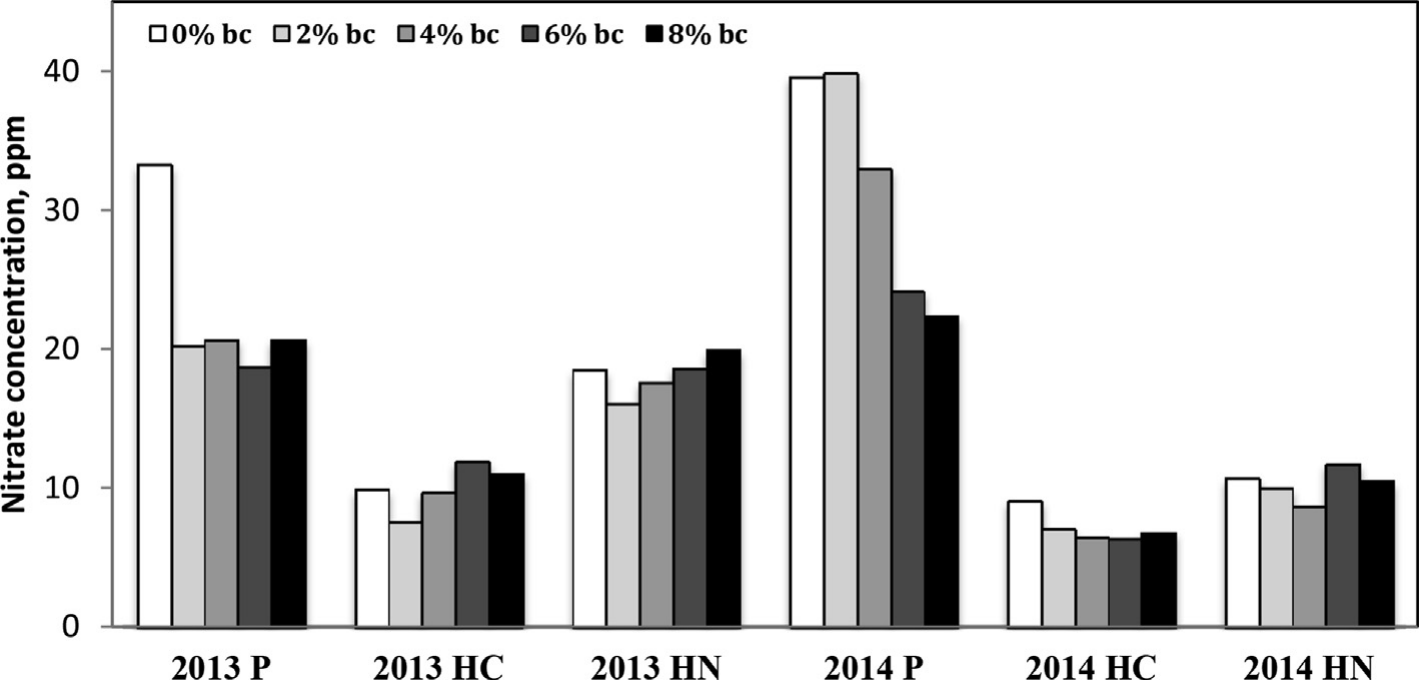
Total soil nitrate concentration at time of harvest, 0–40cm depth. P is PSNT timing, HC is at time of harvest control (no fertilizer), HN is at time of harvest with nitrogen fertilizer treatment (56kg ha^−1^).

Nitrate content in soils during years 2 and 3 were determined at 0–20, 20–40, and total 0–40 cm depth (Fig. 2, Fig. 3) and were twice each season, at both typical PSNT timing and at harvest of sweetcorn. Sub-plots were fertilized with calcium ammonium nitrate after PSNT soil samples were taken. No significant differences in nitrate concentrations were found, except at PSNT in the 3rd year (2014) where biochar treatments of 4, 6, and 8% were significantly lower than the control and 2% biochar treatments.

**Fig. 4:**
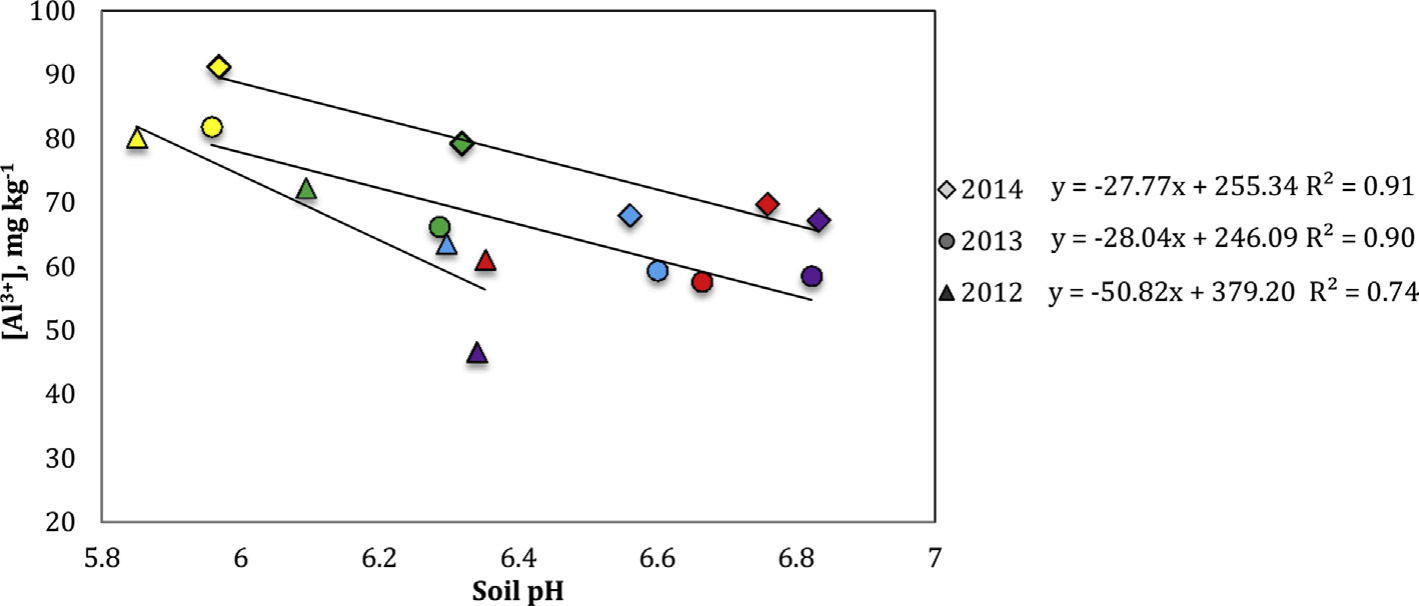
Soil pH at time of harvest and corresponding Al^3+^ concentration. Yellow, green, blue, red and purple correspond to 0, 2, 4, 6 and 8% biochar respectively.

The control soil maintained pH values below 6, whereas the biochar amended soils all had elevated soil pH, increasing throughout the experiment and maintaining pH values of 6.2–6.8 [1]. It is important to note, that all biochar treatment levels significantly increased the soil pH within the acceptable range for field crop growth. Higher Al^3+^ concentration corresponded to lower application rate of biochar (Fig. 4).

**Fig. 5:**
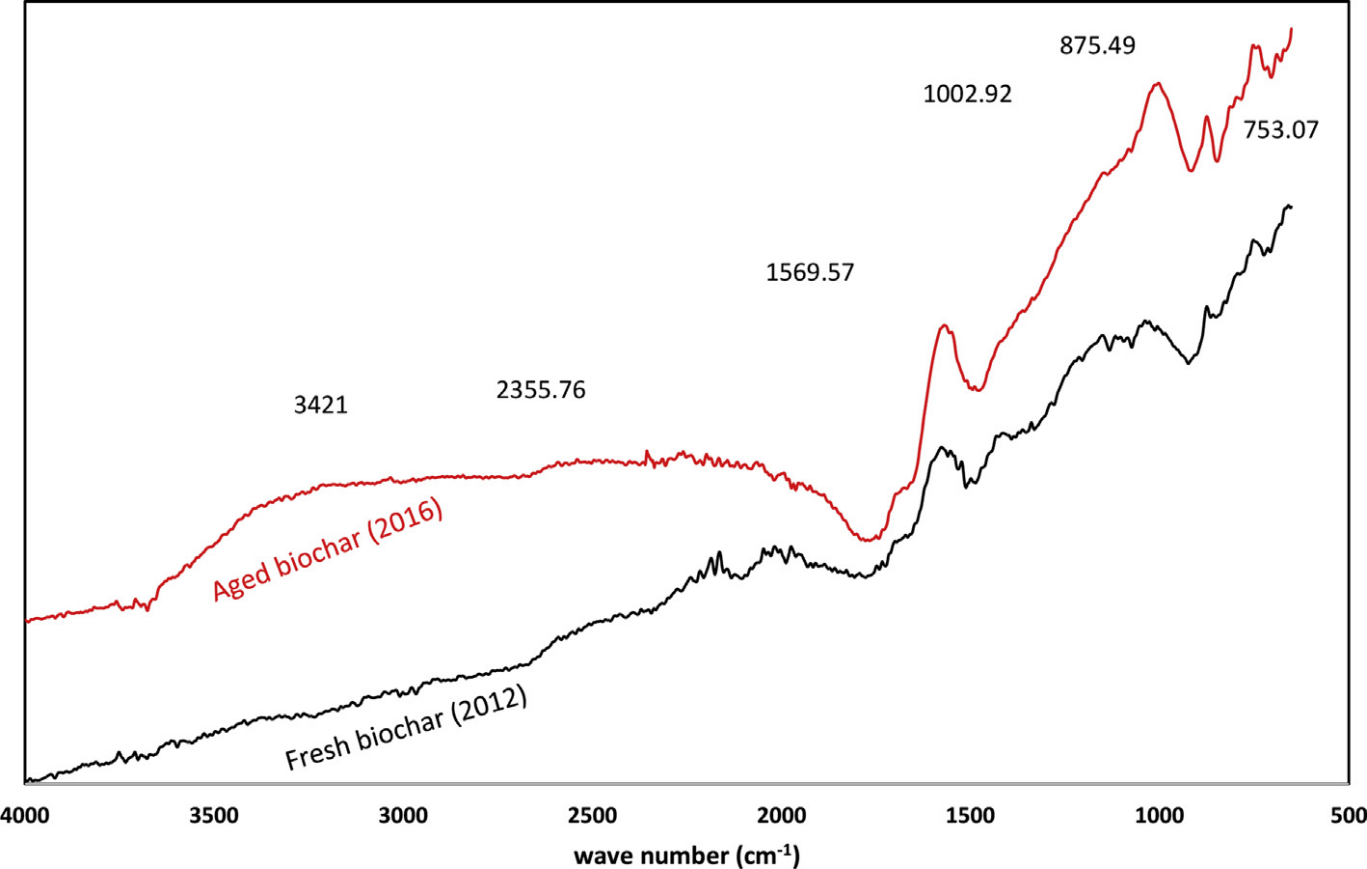
FTIR spectra peaks of fresh and aged sugar maple hardwood biochar.

The FTIR revealed functional group content differences between fresh and aged biochar samples on the content of polar and O-containing functional groups on the surface of biochar (Fig. 5, Table 1). The presence of the new functional groups in aged biochar at 753.07 and 875.49 cm^−1^ can be assigned to C–O–O- stretch from peroxide functional groups (Fig. 5).

**Table 1:** FTIR wave number identification of fresh and aged sugar maple hardwood biochar.

Fresh biochar (2012)	Aged biochar (2016)	Corresponding characteristic vibration	Functional groups	reference
753.07	753.07	=C–H bending (675–1000), C–H out of plane bending (750), O–H out of plane bending (650–770)	Alkene, Aromatic	[10], [11]
875.49	875.49	=C–H bending (675–1000), C–H out of plane bending (830 and 874), C–O–C- stretch (875), γ-CH of furan (875), 1 adjacent H deformation	Aromatic, peroxide	[2], [6], [8], [9], [10]
1038.89	1002.92	C–O stretch (1050–1150), C–O–C symmetric stretching (1097cm^−1^), C–O (1000–1300), C–O–C (1046), C–O–O- (1031), Aromatic C=O stretching (1054–1060)	Aromatic, Alcohol&phenol, aliphatic (Aryl-alkyl ethers),	[8], [9], [10], [11], [12]
1577.01	1569.57	C=C and C=O stretching (1600-1700 cm^−1^), C=C stretch (1400–1600), Aromatic skeletal vibration with C=O stretching vibration (1597), C=O (1587), asymmetric stretching vibrations of COO- (1560),	Aromatic, benzene ring	[8], [10], [11], [12]
2018.32	2355.76	-C=-C stretch (2100–2260), CO2 adsorption (2332), Carbonyl bond group	Alkyne	[6], [7]
aND	3400	O–H (3200–3550), O–H stretch (3428–3437),	Carboxylic acid or water adsorption	[5]

a ND (Not Detected).

## 2 Experimental design, materials and methods

### 2.1 Soil gravimetric moisture content

Soil samples were collected and tested for variations in the total moisture content. Samples were collected 2 days after a rain event. Crucibles were oven dried for 24 hours at 100 °C and weighed before adding soil samples. Crucible weights were recorded with the added soil. Crucibles and soil were then again oven dried at 100 °C for 72 hours and reweighed. Moisture content was then calculated as grams of moisture lost per dry soil weight.

### 2.2 Soil Al/nitrate-N concentration determination

The data on soil Al and nitrate-N concentration is provided in Fig. 2, Fig. 3, Fig. 4 respectively. Soil samples were extracted in 40 mL of Modified Morgan Extractant and shaken at 200 oscillations per minute for 15 minutes, as recommended by the North East Soil Testing Laboratory Manual. Samples were filtered using medium grade filter paper and diluted 1:5 with deionized H_2_O. Samples were analyzed for nitrate-N using flow injection analysis (QuickChem 8000, by LaChat Instruments, Loveland, CO [14]). Samples from the above soil filtrates were then analyzed for Al using the microwave plasma atomic emission spectrophotometry MP-AES Agilent 4100 (Agilent Technologies, Santa Clara, CA; [4], [13]).

#### 2.2.1 FTIR

The infrared spectra (FTIR) were recorded from pellets containing 2 mg of the air-dried biochar. The surface functional groups of fresh and aged biochar samples were identified using a PerkinElmer Spectum One spectrometer with ATR attachment. IR spectra were collected from 4000 to 650 cm^−1^ with a resolution of 2 cm^−1^. The functional groups were identified according to published references (Table 1).

All data were analyzed by one-way ANOVA using the GLM Procedure in SAS 9.4 (SAS Institute Inc., Cary, NC; [3]).
